# SIRT4 regulates ATP homeostasis and mediates a retrograde signaling via AMPK

**DOI:** 10.18632/aging.100616

**Published:** 2013-11-26

**Authors:** Linh Ho, Allen Sam Titus, Kushal Kr Banerjee, Suji George, Wei Lin, Shaunak Deota, Asish K. Saha, Ken Nakamura, Philipp Gut, Eric Verdin, Ullas Kolthur-Seetharam

**Affiliations:** ^1^ The Gladstone Institute of Virology and Immunology, San Francisco, CA 94158, USA; ^2^ Tata Institute of Fundamental Research, Homi Bhabha Road, Colaba, Mumbai, India; ^3^ The Gladstone Institute of Neurological Disease, San Francisco, CA 94158, USA; ^4^ Boston University, Boston, MA 02118, USA; ^5^ Department of Neurology, University of California, San Francisco, CA 94158, USA; ^6^ Department of Medicine, University of California, San Francisco, CA 94158, USA

**Keywords:** Sirt4, ANT2, AMPK, ATP, mitochondrial signaling

## Abstract

Efficient coupling of cellular energy production to metabolic demand is crucial to maintain organismal homeostasis. Here, we report that the mitochondrial Sirtuin Sirt4 regulates mitochondrial ATP homeostasis. We find that Sirt4 affects mitochondrial uncoupling via the adenine nucleotide translocator 2 (ANT2). Loss of Sirt4 expression leads to decreased cellular ATP levels *in vitro* and *in vivo* while Sirt4 overexpression is associated with increased ATP levels. Further, we provide evidence that lack of Sirt4 activates a retrograde signaling response from the mitochondria to the nucleus that includes AMPK, PGC1α, key regulators of β-oxidation such as Acetyl-CoA carboxylase, and components of the mitochondrial respiratory machinery. This study highlights the ability of Sirt4 to regulate ATP levels via ANT2 and a feedback loop involving AMPK.

## INTRODUCTION

Maintenance of energy balance is vital for cellular and organismal physiology. In addition to affecting cellular functions, deregulated energy homeostasis is associated with metabolic diseases, such as obesity and diabetes. Rates of utilization and metabolic fates of carbohydrates and lipids are coupled to the energy demands of a cell (and an organism). Organismal energy balance is regulated by multiple mechanisms and involves mitochondria, which contain the main intracellular machinery for ATP production. As such, mitochondria are key components that couple metabolic inputs to energy homeostasis. Integration of factors intrinsic and extrinsic to mitochondria affect mitochondrial functions to maintain the balance between metabolic inputs and energy homeostasis [1].

Sirtuins are NAD^+^-dependent enzymes that mediate cellular physiology in response to metabolic inputs and energy demands [2, 3]. While all sirtuins rely on NAD^+^ as a cofactor [4-6], only Sirt1, 2 and 3 exhibit robust deacetylase activity, and Sirt5 and 6 exhibit novel deacylase catalytic activities [7-11]. Three mitochondrial sirtuins, Sirt3, Sirt4 and Sirt5 in mammals, affect cellular and organismal physiology by regulating mitochondrial functions [12, 13]. Sirt4, the evolutionarily conserved mitochondrial sirtuin in metazoans, was initially identified as an ADP-ribosylase [14] and shown to regulate insulin secretion [14, 15]. In addition, its ability to regulate glutamine metabolism via GDH has been shown to be important for cancer development [16, 17]. Sirt4 is a critical regulator of fat metabolism [10, 18]. Sirt4 deficiency leads to increased beta-oxidation, which involves an increased transcription of fatty acid oxidation genes in the nucleus [18]. However, the mechanism by which Sirt4 in mitochondria regulates nuclear transcriptional response is unknown. Recently, Sirt4 was shown to possess deacetylase activity [10] and its ability to deacetylate malonyl-CoA-decarboxylase (MCD) has been implicated in regulating fat metabolism. In agreement with this finding, Sirt4 knockout mice burn more fat and hence are protected from high fat diet-induced obesity [10].

The metabolic switch between carbohydrate and fat is coupled with ATP homeostasis [19, 20]. Specifically, at the mitochondrial level, the transition between fed and starved conditions is regulated by mechanisms that alter carbon chain utilization to match cellular energy demand [19, 20]. Despite this knowledge it is still unclear if Sirt4 has a role in controlling cellular ATP levels as an underlying cause of the described phenotypes of Sirt4 deficiency in cells and animals. Thus, understanding the functions of Sirt4 in coupling metabolic flux and energy homeostasis is of major importance, and could potentially be exploited therapeutically.

AMP-dependent kinase (AMPK) is a central sensor of cellular energy status [21, 22]. During energy-deprived conditions, such as starvation, fat oxidation is increased, and mitochondrial functions are elevated [1, 23]. AMPK phosphorylates and inhibits acetyl-CoA-carboxylase (ACC), leading to a decrease in malonyl-CoA levels, which ultimately results in increased carnitine palmitoyl transferase (CPT1) dependent transport and oxidation of fatty acids in mitochondria [24-26]. AMPK also regulates the expression of nuclear-encoded mitochondrial genes via the transcriptional co-activator peroxisome proliferator-activated receptor gamma coactivator 1-alpha (PGC1α) [20, 22, 23]. AMPK-dependent phosphorylation of PGC1α results in increased transcription of genes involved in fatty acid oxidation and mitochondrial biogenesis [20, 22, 23, 27]. Although AMPK senses AMP/ATP ratios, its role in mediating a retrograde signaling from mitochondria to nucleus (and particularly, in a sirtuin-dependent manner) is poorly appreciated.

In this study, we have investigated the role of Sirt4 in energy homeostasis. Moreover, we establish a functional relationship between SIrt4 and the Adenine nucleotide translocator 2 (ANT2), which is also known to act as an uncoupler [28-30]. Our results describe a Sirt4-dependent molecular mechanism that mediates a retrograde signaling to the nucleus.

## RESULTS

### SIRT4 regulates cellular ATP levels

**Figure 1:**
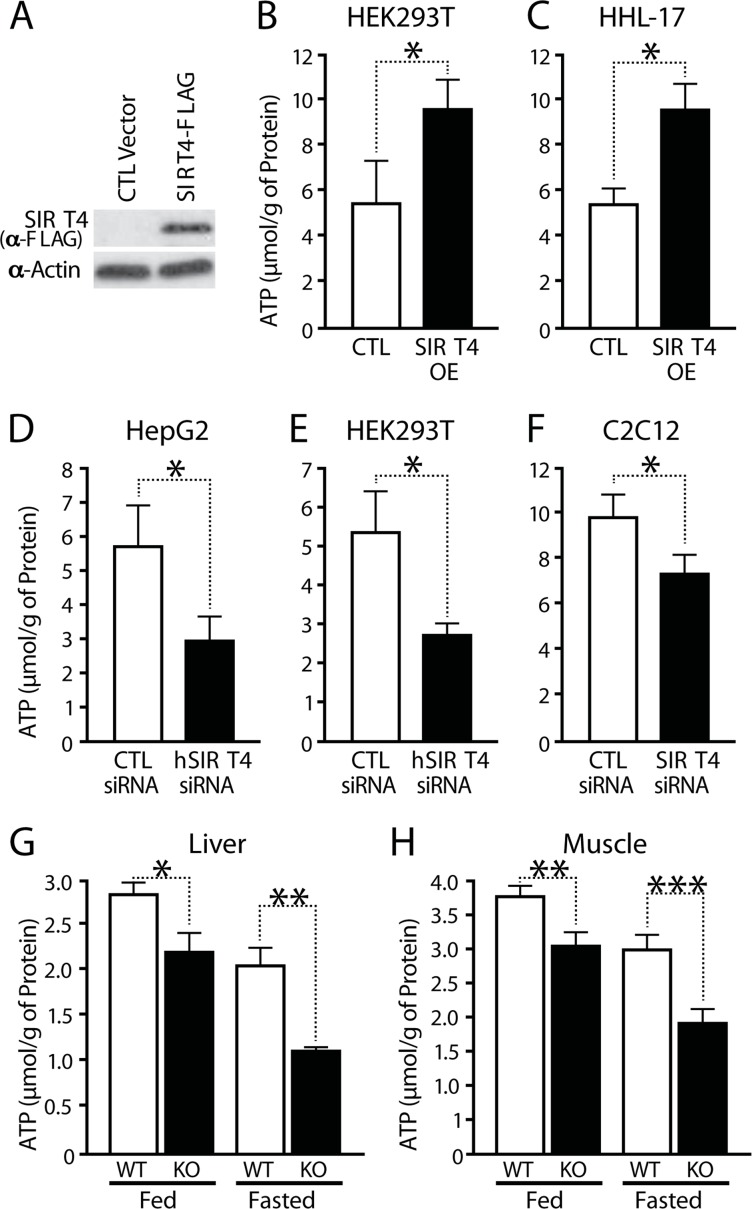
SIRT4 regulates cellular ATP levels (**A**) Western blot showing overexpression of Sirt4. (**B–F**) ATP levels in Sirt4 overexpressing or knockdown cells (HEK293T, HHL-human hepatocytes, HEPG2 and C2C12 myotubes), as indicated. (**G, H**) ATP levels in wild-type and *Sirt4*KO (**G**) liver and (**H**) muscles under fed and fasted conditions. Statistical significance was calculated using student's t-test: * p < 0.05, ** p < 0.01, *** p < 0.001 or as indicated. Error bars indicate mean values ± SEM.

To test whether Sirt4 regulates cellular bioenergetics, we assayed for total ATP in cells after upregulating or downregulating Sirt4 expression in several cell types (Figures 1A-1F and Supplementary Figure S1A). Overexpression of Sirt4 led to an increase in cellular ATP in both HEK293T and HHL-17 cells (Figures 1B and 1C). Conversely, knocking down endogenous Sirt4 led to a marked decrease in total ATP in HepG2, HEK293T and C2C12 myotubes (Figures 1D to 1F and Supplementary Figure 1A).

Next, we tested whether Sirt4 is involved in energy homeostasis *in vivo*. Measurement of total ATP levels in muscles and liver from wild-type and *Sirt4* knockout (*Sirt4KO*) mice showed a significant decrease in ATP content in *Sirt4*KO tissues (Figure 1G, H; Supplementary Figure S1B). Interestingly, lack of Sirt4 led to reductions in ATP under both fed and starved conditions (Figures 1G, H).

The total cellular ATP pool is affected by ATP production from glycolysis and mitochondrial oxidative phosphorylation [19]. To determine if the Sirt4-dependent increase in ATP was due to mitochondrial ATP synthesis, ATP levels were measured in cells treated for 2 hours with 2-deoxy-glucose (2-DG), an inhibitor of glycolysis. Interestingly, the difference in total ATP levels was maintained in 2-DG treated cells (Supplementary Figure S1C). To determine whether this change was secondary to an elevated total nucleotide pool or a change in ATP homeostasis, we measured ADP/ATP ratios. We found that cells overexpressing Sirt4 have a lower ADP/ATP ratio than controls (Supplementary Figure S1D). Conversely, ADP/ATP ratios increased in *Sirt4*KO tissues (Supplementary Figures S1E and S1F). Therefore, these data provide evidence that Sirt4 regulates ATP synthesis within mito-chondria.

### Sirt4 regulates mitochondrial respiration via ANT2-mediated coupling efficiency

**Figure 2:**
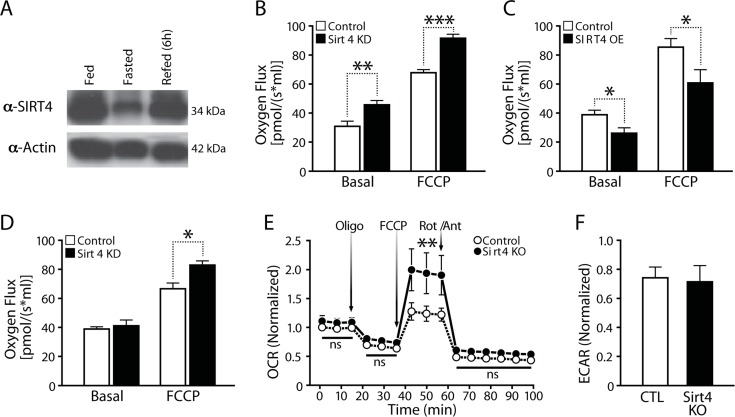
Cellular respiration is negatively associated with Sirt4 expression (**A**) Western blot showing liver Sirt4 expression during fed, fasted and refed conditions. (**B**) and (**C**) Oxygen flux (respiration) in control, Sirt4KD (**B**) and Sirt4OE (**C**) HEK293T cells. (**D**) Oxygen flux (respiration) in control and Sirt4KD HEK293T cells in media supplemented with 2mM pyruvate. (**E**) Oxygen consumption rate (OCR, respiration) in primary hepatocytes isolated from wild-type and *Sirt4*KO mice. OCR was measured under basal and oligomycin, FCCP and Rotenone/Antimycin-A treated conditions (media supplemented with 2mM pyruvate). (**F**) ECAR (glycolysis flux) in primary hepatocytes isolated from wild-type and *Sirt4*KO mice. Oxygen flux was measured under basal and FCCP treated conditions. Statistical significance was calculated using student's t-test: * p < 0.05, ** p < 0.01, *** p < 0.001 or as indicated. Error bars indicate mean values ± SEM.

Net changes of intracellular ATP levels are a function of its production and consumption. Mitochondrial respiration and the coupled flux through the electron transport chain (ETC) drive ATP synthesis. To determine if components of cellular ATP production are regulated by Sirt4, we examined mitochondrial respiration in loss- and gain-of-function experiments. Since endogenous Sirt4 expression was drastically reduced during fasting (Figure 2A), we wanted to assess the effect of Sirt4 perturbations on basal respiration and under conditions that mimick starvation by adding 2mM pyruvate to the culture medium [31]. Transient knockdown of Sirt4 in HEK293T cells led to increased oxygen consumption under basal and FCCP treated conditions (Figure 2B). In contrast, Sirt4 overexpression (*Sirt4*OE) led to decreased oxygen consumption (Figure 2C). Interestingly, knocking down Sirt4 in cells under starvation-like conditions abolished the difference in basal oxygen flux (Figure 2D). In agreement with these findings, basal oxygen consumption was unaffected in primary hepatocytes (media supplemented with pyruvate) isolated from *Sirt4* knockout mice. However, oxygen consumption during FCCP-mediated un-coupling was much greater in *Sirt4*KO mice than in control animals (Figure 2E). The rate of extracellular acidification, which indicates the amount of glycolysis within a cell, remained unchanged (Figure 2F).

To test whether Sirt4 directly affects ATP synthase activity, we measured the activity of immunocaptured ATP-synthase and detected a comparable enzymatic activity in control and *Sirt4*OE cells (Supplementary Figure 1G). Failure to maintain ATP levels despite increased oxygen consumption in Sirt4 deficient cells and the lack of change in ATP-synthase activity indicate that Sirt4 affects the coupling of ETC flux to ATP production and oxidative phosphorylation (OXPHOS) efficiency.

Uncoupling proteins (UCPs) and adenine nucleotide translocator proteins (ANTs) regulate the coupling efficiency of the electrochemical gradient across the inner mitochondrial membrane. Interestingly, ANTs have a dual role in regulating energy homeostasis: they transport ADP-ATP across the inner mitochondrial membrane, and they act as uncoupling proteins. Acylated ANTs uncouple mitochondria and reduce the efficiency of oxidative phosphorylation [30, 32, 33]. Moreover, uncoupling in the liver predominantly depends on ANT2 [34]. Our results show that Sirt4-dependent changes in ATP and mitochondrial respiration correlate negatively.

**Figure 3:**
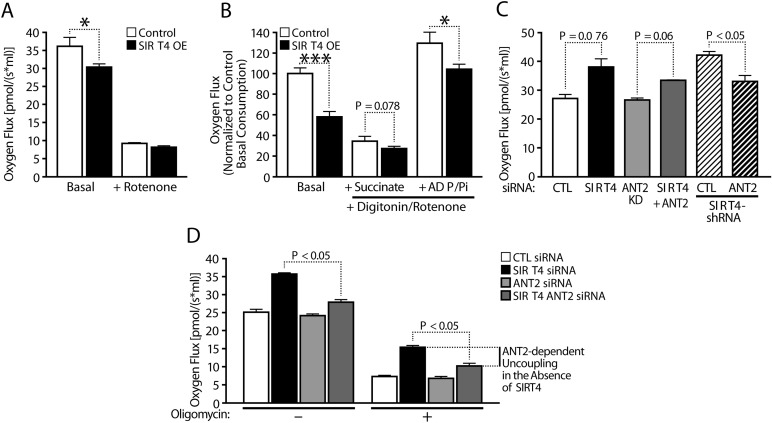
Sirt4 regulates mitochondrial respiration via ANT2-mediated coupling efficiency (**A**) Oxygen consumption in control and Sirt4OE HEK293T cells under basal and Rotenone treated conditions. (**B**) Oxygen consumption in permeabilized control and Sirt4OE HEK293T cells under basal conditions and in response to sequential addition of substrates, succinate and ADP/Pi. Rotenone was added to inhibited complex-I when measuring respiration in response to succinate and ADP/Pi addition, as indicated. (**C, D**) Oxygen consumption in control and Sirt4KD HEK293T cells under basal (**C, D**) and oligomycin treated (**D**) conditions. Sirt4 alone or Sirt4 and ANT2 were simultaneously knocked down to measure ANT2-dependent uncoupled respiration and as indicated. Statistical significance was calculated using student's t-test and ANOVA: * p < 0.05, ** p < 0.01, *** p < 0.001 or as indicated. Error bars indicate mean values ± SEM.

Since we previously reported that Sirt4 interacts with endogenous ANT2 [15], we tested whether Sirt4 affects the coupling efficiency of oxidative phosphorylation in an ANT2-dependent manner and determined whether Sirt4-dependent changes in oxygen consumption are due to coupling efficiency. The reduced oxygen flux in *Sirt4*OE cells was lost when cells were treated with Rotenone (inhibitor of Complex-I) (Figure 3A). Further, the difference between control and *Sirt4*OE cells was retained when permeabilized cells were provided with succinate and ADP/Pi (Figure 3B). This difference in oxygen consumption (when succinate was provided as a substrate) also ruled out possible effects from alterations in Sirt4 mediated and GDH-dependent changes in TCA flux via α-ketoglutarate (α-KG).

Importantly, the decrease and increase in oxygen consumption observed in *Sirt4*KD and in *Sirt4*OE cells was insensitive to Oligomycin treatment (ATP synthase inhibitor) (Figure 3C, D; Supplementary Figure 3). Based on these results, we conclude that Sirt4 regulates coupling efficiency.

Next, we tested whether the increased uncoupling in Sirt4KD cells was mediated by ANT2. As shown earlier, knocking down Sirt4 led to increased oxygen consumption (Figure 3C, D). Interestingly, down-regulation of ANT2 in these cells suppressed the basal rate of respiration (Figure 3C, D). Importantly, the oxygen flux between *Sirt4*KD and *Sirt4*KD/*ANT2*KD was significantly different post-Oligomycin treatment (Figure 3D). Knocking down ANT2 along with Sirt4 restored oxygen consumption to control levels (Figure 3D). These results indicate that Sirt4 regulates coupling efficiency in an ANT2-dependent manner.

### SIRT4 regulates ATP in an ANT2-dependent manner

**Figure 4:**
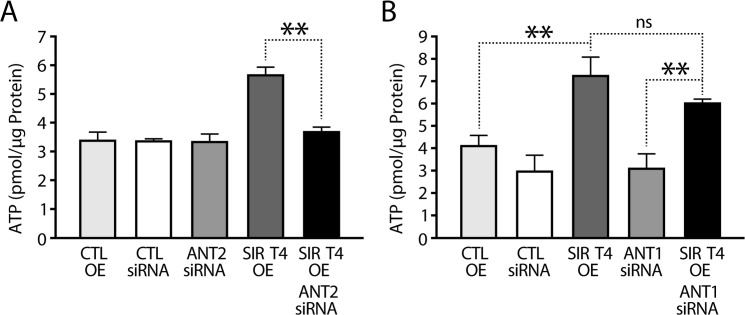
SIRT4 regulates ATP in an ANT2-dependent manner (**A**) ATP levels in HEK293T cells that were transfected with control, ANT2-siRNA, Sirt4-FLAG and Sirt4-FLAG/ANT2-siRNA constructs, as indicated. # indicates p < 0.05 for cells transfected with Sirt4-FLAG in the presence or absence of ANT2 (+/− ANT2-siRNA). (**B**) ATP levels in HEK293T cells that were transfected with control, ANT1-siRNA, Sirt4-FLAG and Sirt4-FLAG/ANT1-siRNA constructs, as indicated. Statistical significance was calculated using student's t-test and ANOVA: * p < 0.05, ** p < 0.01, *** p < 0.001 or as indicated. Error bars indicate mean values ± SEM.

The results presented above prompted us to investigate whether Sirt4-ANT2 interaction was required for maintaining cellular ATP levels. As shown earlier, we detected elevated ATP levels in *Sirt4*OE cells. Importantly, this increase was reduced to control levels when ANT2 was knocked down simutaneously (Figure 4A and Supplementary Figure S2A).

ANT protein family members are expressed in a tissue-specific manner and may have different functions [30, 35]. While mice have two proteins (ANT1 and ANT2), humans have four (ANT1–4) [35]. Of these four, the functions of ANT1 and ANT2 are well characterized. To check if the ability of Sirt4 to affect ATP homeostasis was restricted to its interaction with ANT2, we knocked down ANT1 in Sirt4-overexpressing HEK293T cells (Supplementary Figure S2B). In contrast to what we had seen with ANT2 knockdown, downregulation of ANT1 did not reduce Sirt4-mediated increase in total ATP (Figure 4B). Importantly, only ANT2 is expressed in liver (Supplementary Figure S2D) [34, 35], and these results demonstrate the specificity of Sirt4-ANT2 interaction in energy metabolism.

### Reduction of ATP levels by Sirt4 deficiency initiates a homeostatic feedback loop via ANT2/AMPK

**Figure 5:**
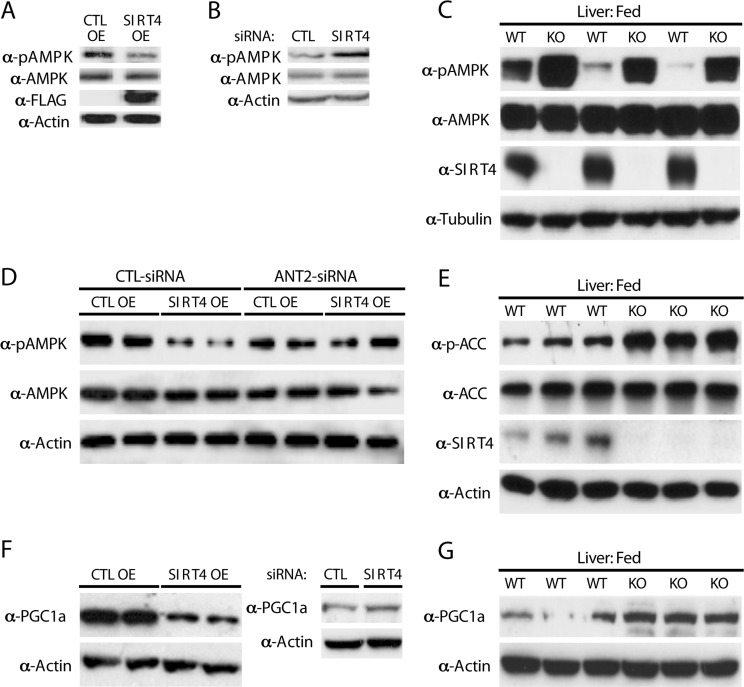
Sirt4 deficiency initiates a homeostatic feedback loop via ANT2/AMPK (**A**) Western blots for p-AMPK, AMPK, Sirt4-FLAG and actin in control and Sirt4OE HEK293T cells. (**B**) Western blots for p-AMPK, AMPK and actin in control and Sirt4KD HEK293T cells. (**C**) Western blots for p-AMPK, AMPK, Sirt4 and tubulin in liver lysates from wild-type and *Sirt4*KO mice under fed conditions. (**D**) Western blots for p-AMPK, AMPK and actin in control and Sirt4OE HEK293T cells in the presence or absence of ANT2 (+/− ANT2-siRNA), as indicated. (**E**) Western blots for p-ACC, ACC, Sirt4 and actin in liver lysates from wild-type and *Sirt4*KO mice under fed conditions. (**F**) Western blots for PGC1α and actin in control and Sirt4OE or Sirt4KD HEK293T cells, as indicated. (**G**) Western blots for PGC1α and actin in liver lysates from wild-type and *Sirt4*KO mice under fed conditions.

Sensing and signaling of cellular energetic status depends on the activity of AMPK, which is allosterically activated by AMP/ADP [22, 23, 27]. Since Sirt4 activity regulates ATP levels, we addressed whether AMPK activity was affected. Importantly, overexpression of Sirt4 led to a marked decrease in pAMPK levels (Figure 5A). Conversely, knocking down Sirt4 resulted in an increase in AMPK activity (Figure 5B). In agreement with these observations, measurement of pAMPK *in vivo* showed a strong increase in its levels in livers of mice lacking SIRT4 in comparison to littermate controls (Figure 5C).

Notably, the decrease of pAMPK in *Sirt4*OE cells was rescued when ANT2 was simultaneously knocked down, demonstrating that the cross-talk between Sirt4 and AMPK requires ANT2 (Figure 5D). These results establish a novel functional interaction between Sirt4 and AMPK and its dependency on ANT2.

Increased fatty acid oxidation during fasting depends on AMPK activity. AMPK-mediated phosphorylation of acetyl-CoA-carboxylase (ACC) leads to decreased malony-CoA levels, derepression of CPT1 [24-26] and increased mitochondrial fatty acids uptake [24, 36]. Since Sirt4 has been implicated in the regulation of fatty acid metabolism [10, 18], we tested the effect of Sirt4 on ACC phosphorylation. In agreement with the observed increase in AMPK activity, we found that mice lacking Sirt4 showed increased phosphorylation of ACC (Figure 5E).

In addition to modifying metabolic enzymes, AMPK also regulates the transcriptional coactivator PGC1α. We found that PGC1α protein levels were inversely correlated with Sirt4 in cells (Figure 5F). Induction of PGC1α was also observed in liver from *Sirt4*KO mice (Figure 5G). Treating *Sirt4*OE cells with AICAR (an AMPK activator) rescued the decrease of both pAMPK and PGC1α (Supplementary Figure S4). These results support the model that Sirt4 and AMPK interact via ANT2 to mediate a retrograde signaling to ACC and PGC1α.

### Sirt4-AMPK mediated retrograde signaling regulates expression of fatty acid oxidation genes

**Figure 6:**
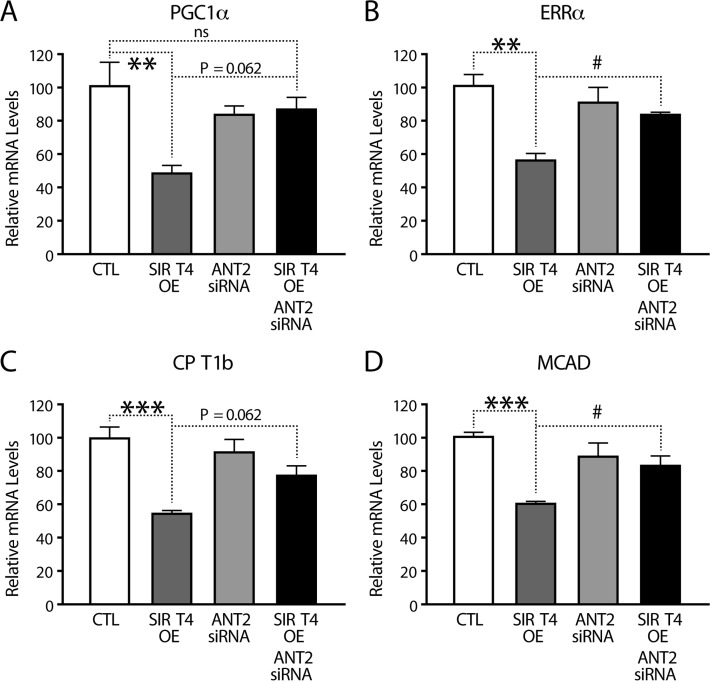
Sirt4-AMPK mediated retrograde signaling regulates FAO gene expression (**A–D**) Quantitative RT-PCR of genes involved in fatty acid oxidation (**A**) *PGC1α*, (**B**) *ERRα*, (**C**) *CPT1b* and (**D**) *MCAD* from RNA isolated from HEK293T cells transfected with control, Sirt4-FLAG, ANT2-siRNA and Sirt4-FLAG/ANT2-siRNA constructs. Statistical significance was calculated using ANOVA: * p < 0.05, ** p < 0.01, *** p < 0.001 or as indicated. Error bars indicate mean values ± SEM.

PGC1α plays an important role in the fasting response by activating transcription of fatty acid oxidation (FAO) genes [37, 38]. Having established a retrograde signaling from Sirt4 to PGC1α via ANT2 and AMPK, we measured the transcription of FAO genes. As predicted, PGC1α and its downstream FAO genes (such as *ERRa, CPT1b* and *MCAD*) were significantly downregulated in *Sirt4*OE cells than in controls (Figures 6A–D). Importantly, knocking down ANT2 in these cells rescued the downregulation and restored their expression.

### Sirt4 regulates mitochondrial biogenesis in a feedback loop via ANT2/AMPK/PGC1α signaling

The cellular response to restore energy homeostasis includes mitochondrial biogenesis, which is often seen during exercise, fasting or mild uncoupling [20]. Increased expression of nuclear-encoded mitochondrial genes by AMPK-PGC1α regulates mitochondrial mass under these conditions [20, 23]. Transcription of genes, such as *PGC1α, ERRα, TFAM, NRF1* and *Cyt.C*, is a hallmark of mitochondrial biogenesis [20].

**Figure 7:**
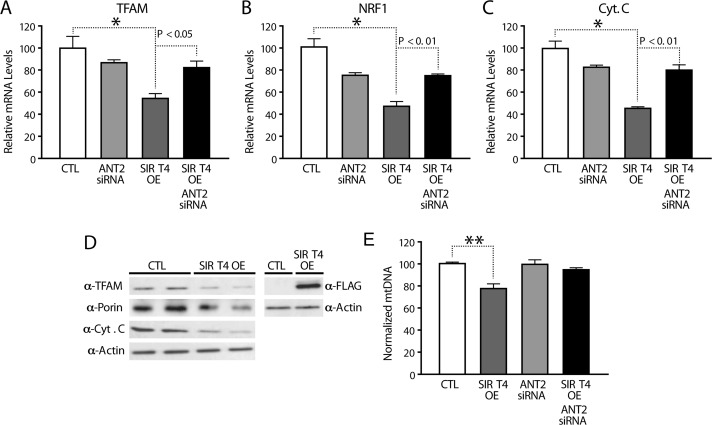
Sirt4 regulates expression of nuclear encoded mitochondrial genes in a feedback loop via ANT2/AMPK/PGC1α signaling (**A–C**) Quantitative RT-PCR of nuclear encoded mitochondrial genes (**A**) *TFAM*, (**B**) *NRF1* and (**C**) *Cytochrome C* from RNA isolated from HEK293T cells transfected with control, Sirt4-FLAG, ANT2-siRNA and Sirt4-FLAG/ANT2-siRNA constructs. (**D**) Western blots for TFAM, Porin, Cytochrome C, Sirt4-FLAG and actin in control and Sirt4OE HEK293T cells. (**E**) Quantitative RT-PCR of mitochondrial DNA (normalized to nuclear DNA) in DNA isolated from HEK293T cells transfected with control, Sirt4-FLAG, ANT2-siRNA and Sirt4-FLAG/ANT2-siRNA constructs. Statistical significance was calculated using ANOVA: * p < 0.05, ** p < 0.01, *** p < 0.001 or as indicated. Error bars indicate mean values ± SEM.

Measurement of expression of these genes showed lower mRNA levels in *Sirt4*OE cells than in controls (Figure 7A–C). Knocking down ANT2 in *Sirt4*OE cells rescued the decreased mRNA levels of *PGC1α, ERRα, TFAM, NRF1*, and *Cyt.C* (Figures 7A–C and 6B). This finding further strengthens an important role of the Sirt4-ANT2 interaction in mediating a compensatory change in the expression of nuclear-encoded mito-chondrial genes.

In agreement with these changes, we also found a significant decrease in the protein levels of TFAM and cytochrome C in both HEK293T (Figure 7D) and HEPG2 (Supplementary Figure 5) upon Sirt4 over-expression. We also observed a small but notable effect on Porin levels (Figure 7D). Importantly, mitochondrial DNA (mtDNA), a marker of mitochondrial biogenesis, was decreased in *Sirt4*OE cells and this was suppressed in cells lacking ANT2 (Figure 7E). Our data indicate that mitochondrial Sirt4 controls key regulators of mitochondrial mass as well as the transcription of OXPHOS components via a feedback regulatory loop involving ANT2-AMPK-PGC1α.

## DISCUSSION

The results presented above support an important role for Sirt4 in energy homeostasis. Specifically, its ability to alter cellular ATP requires the adenine nucleotide translocator 2 (ANT2), a transmembrane protein located on the inner mitochondrial membrane. Furthermore, we show that ANT2-mediated oxidative phosphorylation (OXPHOS) efficiency is regulated by Sirt4 and loss of Sirt4 leads to increased oxygen consumption by uncoupling. Sirt4 deficiency that mimics energy deprived condition initiates a homeostatic response involving AMPK and PGC1α. We propose that Sirt4 controls the efficiency of ATP production. Further, integration of mitochondrial signaling with nuclear transcription couples fluctuations in cellular energy needs with metabolic inputs. In this regard, we have uncovered a previously unknown function of Sirt4 and show that it establishes a retrograde signaling to the nucleus to maintain energy homeostasis.

By overexpressing and knocking down Sirt4 as well as using *Sirt4* knockout animals to study this gene *in vivo*, we define its role in energy homeostasis. Importantly, lack of Sirt4 leads to a decrease in ATP levels both in cells and in tissues from *Sirt4*KO mice. Sirt3 has been reported to increase ATP production by deacetylating ETC components and increasing cellular respiration [12, 39-41]. Our results clearly indicate that, in contrast to Sirt3, Sirt4-dependent increase in cellular ATP is not due to increased respiration. To dissect out the mechanism of Sirt4-dependent ATP changes, we investigated the role of ANT2 as it was previously reported to interact with Sirt4 [15]. ANTs are ADP/ATP translocators with various expression and functions in different tissues [30, 35]. Although, ANT1 and ANT2 are evolutionarily conserved, ANT2 is more widely expressed [34, 35]. By knocking down ANT2 in cells that overexpressed Sirt4, we established that Sirt4 requires ANT2 to affect cellular ATP. We also found that, while Sirt4-ANT2 interplay was required for ATP homeostasis, ANT1 did not seem to play a role in Sirt4-dependent changes.

While Sirt4 positively influenced ATP levels, it was negatively correlated with respiration. Dissipation of the electrochemical gradient by proton leak (or uncoupling) leads to increased respiration and also results in decreased ATP production. Interestingly, ANTs have dual functions in mitochondria with regards to energy homeostasis [30, 32, 42]. While they play a critical role as antiporters for ADP and ATP, they uncouple the mitochondrial membrane upon acylation [30, 32, 33]. Thus, we hypothesized that Sirt4 deficiency leads to an ANT2-dependent increase in respiration due to uncoupling. By providing excess substrates (succinate and ADP/Pi), we show that the Sirt4 dependent changes in oxygen consumption are not due to alterations in TCA flux. Importantly, we found that the increased respiration in Sirt4 knockdown cells was lost when ANT2 expression was downregulated. These results suggest that ANT2 could be a potential substrate for Sirt4. Sirt4 exhibits both ADP-ribosylase and deacetylase activities [9, 10]. Although ANT2 might be ADP-ribosylated [43], its activity as an uncoupler has been largely attributed to acylation [30, 32, 33]. It will be interesting to investigate whether Sirt4 possesses a deacylase activity similar to what has been reported for Sirt5 [39]. However, by identifying the nexus of ANT2 and Sirt4, we added a new component to the understanding of the complex biology of Sirt4.

We showed that Sirt4 modulates AMPK activity and is part of a retrograde signaling pathway from the mitochondria to the nucleus. There was a significant increase in pAMPK levels in the absence of Sirt4 (*Sirt4*KD cells and *Sirt4*KO tissues), which was consistent with energy deficit. Importantly, the decrease in AMPK activity in Sirt4 overexpressing cells was rescued when ANT2 was knocked down demonstrating the crucial role of ANT2 in mediating a Sirt4-dependent signal to AMPK. Energy homeostasis is intricately linked to alterations in metabolic flux and mitochondrial functions. Our results support the hypothesis that the inputs from mitochondria via AMPK activity restore energy balance.

The increased AMPK-dependent phosphorylation of ACC that we observed in *Sirt4*KO mice highlights the physiological significance of Sirt4-AMPK signaling in fatty acid oxidation. Specifically, AMPK-dependent pACC-mediated changes in cytosolic malonyl-CoA are critical for regulating CPT1 (on the outer mitochondrial membrane) mediated fatty acid transport [24, 36]. Interestingly, the lean phenotype displayed by Sirt4 deficient mice was shown to be due to increased activity of Malonyl-CoA-Decarboxylase (MCD) in the mitochondria [10]. Our observations demonstrate that the other side of the reaction, mediated via acetyl-CoA carboxylase, is also regulated by SIRT4 via its activation of AMPK.

Finally, we tested the effect of Sirt4-AMPK-mediated retrograde signaling on PGC1α dependent transcription of FAO genes [37, 38]. Notably, in the absence of Sirt4, AMPK-dependent signaling increased the expression of PGC1α and its downstream FAO genes. In agreement with the effects on AMPK activity, reduced expression of *PGC1α, MCAD, CPT1b* and *ERRα* in Sirt4-overexpressing cells were rescued by ANT2 knockdown. These results demonstrate that Sirt4 in the mitochondria not only regulates mitochondrial uptake of fatty acids via AMPK-ACC, but it also controls fatty acid oxidation through AMPK-PGC1α signaling to the nucleus.

Our findings show that Sirt4-AMPK-PGC1α signaling affects mitochondrial biogenesis (transcription of OXPHOS components) in an ANT2-dependent manner. We provide conclusive evidence to suggest that Sirt4 in the mitochondria creates a feedback loop to regulate mitochondrial homeostasis. In response to calorie restriction, starvation and mild uncoupling, AMPK-PGC1α signaling regulates mitochondrial biogenesis [20]. Although, transcriptional regulation of nuclear encoded genes has been extensively studied [20], the ability of mitochondria to provide an instructive cue to control mitochondrial mass and functions is poorly understood [1, 20].

**Figure 8:**
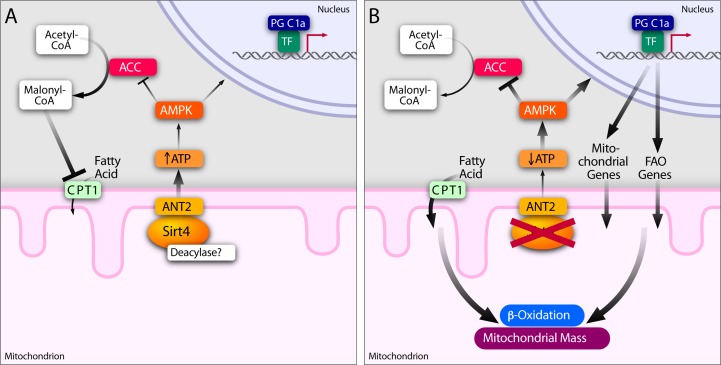
Sirt4-ANT2 interplay regulates energy homeostasis and mediates a retrograde signaling from mitochondria Sirt4-ANT2 interaction in the mitochondria is required for cellular ATP homeostasis. Sirt4-dependent increase in ATP reduces AMPK activity to mediate a retrograde signaling to affect ACC and PGC1α functions. In Sirt4 deficient conditions, ANT2-dependent uncoupling results in a reduction in cellular ATP levels and activates AMPK. Since acylation of ANT2 is known to uncouple mitochondria, we speculate that Sirt4 possesses a deacylase activity. AMPK activation in the absence of Sirt4 consequently leads to phosphorylation of ACC (inhibitory effect), which is known to reduce malonyl CoA levels in the cytosol and thus increase mitochondrial fatty acid uptake (via de-repression of CPT1). In addition, AMPK activation in the absence of Sirt4 increases the expression of PGC1α and its downstream targets involved in beta-oxidation and mitochondrial biogenesis. Together, we propose that Sirt4 in the mitochondria mediates a feedback control to regulate fatty acid metabolism and OXPHOS components.

We found that mitochondrial Sirt4 mediates retrograde signaling. Crosstalk between the NAD^+^-dependent mitochondrial factor, Sirt4, and an AMP/ADP sensor in the cytoplasm/nucleus (AMPK) orchestrates cellular physiology (Figure 8). Retrograde signaling from mitochondria has been well studied in yeast and in plants [44-46]. Although, ATP and calcium dependent signaling are thought to be important in mitochondrial signaling, it is poorly addressed in mammals [44-46]. Therefore, it is interesting to note that the NAD+-dependent Sirt4 in the mitochondria provides instructive cues to alter cellular physiology in addition to regulating a feedback for mitochondrial functions.

To conclude, an inability to utilize fatty acids has been implicated in the onset and progression of metabolic diseases, such as obesity and diabetes. Efforts to increase fatty acid oxidation, for example by activating AMPK, have been only partially successful because of a lack of concomitant alteration in the energy status of a cell [22, 47]. In fact, a simultaneous increase in fatty acid oxidation and energy dissipation (either through exercise or mitochondrial uncoupling) has been speculated to be critical in providing a therapeutic intervention [47-49]. Thus, our report that highlights the central role of Sirt4 in regulating OXPHOS efficiency, ATP homeostasis and fatty acid oxidation could also have important clinical relevance.

## METHODS

### Antibodies and Reagents

Antibodies used were specific for monoclonal and polyclonal AMPKα, Phospho-AMPKα (Thr172), Acetyl-CoA Carboxylase (ACC), Phospho-ACC (Ser79) (Cell Signaling Technology), PGC1α (Millipore and Sant Cruz), SIRT4 as a generous gift from Marcia Haigis [as described [14], FLAG-M2, MYC, Tubulin and β-Actin (Sigma Aldrich). M199 (for culturing primary hepatocytes),. Oligomycin, FCCP, Rotenone and XF Assay medium (Seahorse Bioscience or Sigma Aldrich), AICAR, pyruvate and 2-deoxy glucose (Sigma Aldrich). Fetal bovine serum and Horse serum (Invitrogen), and DMEM (Sigma Aldrich).

### Animal Studies

All animal studies were performed using IACUC-approved protocols. Studies used male WT littermate and SIRT4KO 129Sv mice [14], at 10-14 weeks of age, maintained on a standard chow diet (5053 PicoLab diet), unless otherwise indicated. Mice were sacrificed at 7:00 h for fed mouse studies, or transferred to a new cage without food for 18 h (O/N), and then sacrificed for fasted mouse studies.

### Cell Culture and treatments

HEK293T, HepG2, C2C12 and HHL-17 [50] cells were grown in DMEM supplemented with 10%FBS containing 5mM (low) or 25mM (high) glucose. Confluent C2C12 cells differentiated into myotubes using 1% horse serum. Cells were grown in high glucose medium unless otherwise mentioned. Transfected cells were treated with 20mM 2-DG for 2 hours to transiently inhibit glycolysis and 500μM AICAR for 16 hours for AMPK activation. Cells treated with PBS or DMSO were used as controls.

### Transfection

For Sirt4 and ANT2 overexpression, cells were either stably transfected and selected on puromycin or transiently transfected. Empty pBABE-puro or pcDNA3.1 were used for control transfection. 20nM (unless mentioned otherwise) of scrambled and Sirt4 siRNAs were transfected in HEK293T and 50nM in C2C12. Lipofectamine 2000 (Invitrogen) was used for transfections as per manufacturer's instructions.

### ATP and ADP/ATP ratio measurement

ATP was measured spectrophotometrically as described previously [51] using the method of Oliver H and Lowry JVP (A flexible system of enzymatic analysis, Academic Press, 1972, p 151-153). From cells total ATP and ADP/ATP were determined using ATP bioluminescence assay mix (Sigma Aldrich) and ADP/ATP kits (Mitosciences), respectively.

### Immunoblot analysis

Organs were harvested and homogenized using a tissue homogenizer. Cell and tissue lysates were prepared in ice-cold RIPA buffer (50 mM Tris [pH 7.4], 0.1% SDS, 0.5% sodium deoxycholate, 150 mM NaCl, 1% NP40) at 4°C containing protease inhibitors (EDTA-free Complete; Roche Molecular Biochemicals)), halt or PHOSTOP phosphatase inhibitor cocktail (Thermo scientific and Roche). The lysates were clarified by centrifugation for 15 min at 4°C at 14,000 rpm and protein concentrations were estimated by BCA assay. The lysates were resolved on SDS-PAGE gel, electrophoretically transferred, immunoblotted and signal was detected using ECL (Amersham Biosciences, Thermo Scientific or Roche).

### Plasmids, siRNA and Primers

Human Sirt4 and ANT2 cDNAs were cloned into pBABE-puro and pCDNA3.1 vectors, respectively. Scrambled and Sirt4 siRNAs were obtained from Imagenes and Sigma Aldrich. ANT1 and ANT2 siRNAs were obtained from Sigma Aldrich. List of primers used for cloning is provided in Supplementary table 1.

### RNA isolation and quantitation

Total RNA was isolated using Trizol reagent (Invitrogen) and reverse transcribed using random hexamers and SuperScript-III kit (Invitrogen). Real-time PCR was carried out using primer pair listed in Supplementary table 1 using Eppendorf or Roche real-time machines, and SYBR kit (Kapa Biosystems).

### Oxygen consumption

For primary hepatocytes from SIRT4KO and WT littermates: Oxygen consumption rates (OCR) and extracellular acidification rates (ECAR) were measured in XF assay media (nonbuffered DMEM containing 25 mM glucose, 2 mM L glutamine, and 2 mM sodium pyruvate) under basal conditions and in response to 1 uM oligomycin, 0.5 uM fluoro-carbonyl cyanide phenylhydrazone (FCCP), and 2 uM rotenone with the Seahorse XF-96 Extracellular Flux Analyzer (Seahorse Bioscience). The XF-96 measures the concentration of oxygen and free protons in the medium above a monolayer of cells in real-time. Protein concentration was determined for each well using a standard BCA protein assay. OCR and ECAR values are normalized to mg/protein and are plotted as the mean +/− standard deviation. For cultured cells: Cells were trypsinized and oxygen consumption flux was determined using OROBOROS high resolution respirometer in DMEM (with 25 mM glucose and 2 mM L glutamine with or without 2mM pyruvate) under basal and in response to 2.5μM rotenone, 10mM succinate, 1mM ADP, 1mM orthophosphate (Pi), 0.5μM FCCP and 1μg/mL oligomycin. Two million cells in a total volume of 2.1ml were used for the assays.

### ATP synthase activity assay

Complex-V enzyme activity was measured with ATP synthase enzyme activity microplate assay kit (Mitosciences) according to the manufacturer's instruction. Briefly, the ATP synthase enzyme, from equal amounts of mitochondria, isolated from cells (as described above), was immunocaptured within the wells of the microplate and the enzyme activity was measured.

### Estimation of mtDNA

Total genomic DNA was isolated using the genomic DNA isolation kit (Bangalore Genei/Merck). The relative mitochondrial DNA (mtDNA) content was quantified by qRT-PCR using primers for *Cytochrome b (*mitochondrial genomic DNA) and nuclear *beta Actin* (for nuclear genomic DNA).

### Data processing and Statistical Analyses

Student's T-test and ANOVA were used for statistical analysis (p value: * < 0.05; ** < 0.01; *** < 0.001 or as indicated). Microsoft Excel was used for data processing, and statistical significance was calculated using Excel or Sigmaplot. Results are given as the mean ± standard error. All experiments were performed at least three times

## SUPPLEMENTARY INFORMATION


