# Supplementary material for: SIRT4 regulates ATP homeostasis and mediates a retrograde signaling via AMPK
Source: Aging (Albany NY). 2013 Nov 26;5(11):835–49. doi: 10.18632/aging.100616 (PMC3868726; doi:10.18632/aging.100616)
Supplement: Supplementary file 1 [file aging-05-835-s001.pdf]

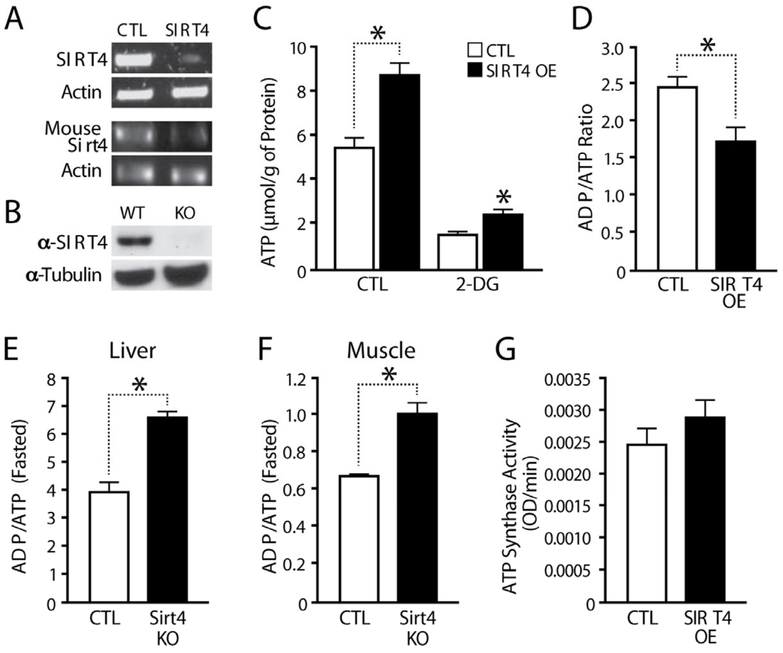

Supplementary Figure 1. (**A**) RT-PCR to show the efficiency of Sirt4 knockdown. (**B**) Western Blot using anti-Sirt4 antibodies in control and Sirt4 knockout mice. (**C**) Total ATP levels in control and Sirt4-overexpressing HEK293T cells, and in response to 2-deoxyglucose treatment. (**D-F**) ADP/ATP ratios in (**D**) control and Sirt4-overexpressing HEK293T cells, (**E**) control and Sirt4-knockout liver under fasting conditions and (F) control and Sirt4-knockout muscles under fasting conditions (**G**) ATP synthase activity in control and Sirt4-overexpressing HEK293T cells.

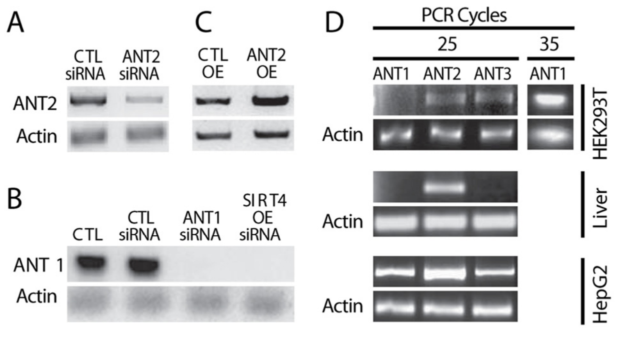

Supplementary Figure 2. (**A-B**) RT-PCR to show knockdown of (**A**) ANT2, (**B**) ANT1 in HEK293T cells. (**C**) RT-PCR to show ANT2 overexpression in HEK293T cells. (**D**) RT-PCR to show differential expression of ANT1, ANT2 and ANT3 in HEK293T cells, HepG2 cells and liver samples.

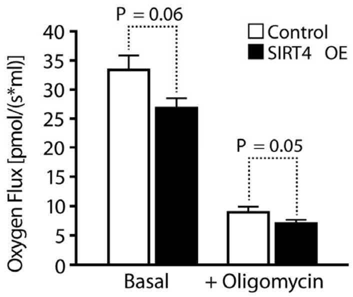

Supplementary Figure 3. Oxygen flux in control and Sirt4-overexpressing HEK293T cells in response to oligomycin treatment.

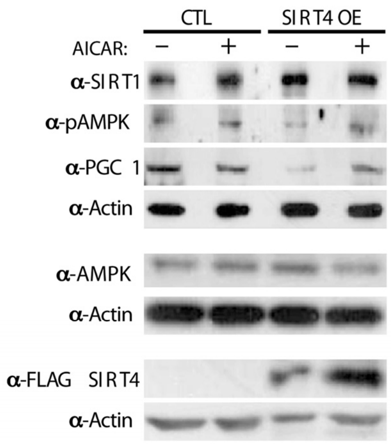

Supplementary Figure 4. Western blot to show the protein levels of Sirt1, phospho-AMPK, Sirt4-FLAG, PGC1α and AMPK in control and Sirt4-overexpressing HEK293T cells, and in response to AICAR treatment.

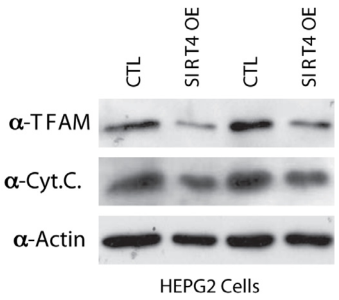

Supplementary Figure 5. Western blot to show the protein levels of TFAM and Cytochrome-C in control and Sirt4-over-expressing HepG2 cells.

Supplementary Table 1. List of primers used for RT-qPCR

ACTIN	F: GCATGGGTCAGAAGGATTCC<br>R: ACGCAGCTCATTGTAGAAGG
CPT1b	F: TCGTCACCTCTTCTGCCTTT<br>R: ACACACCATAGCCGTCATCA
PGC1α	F: GTGGATGAAGACGGATTGCC<br>R: GCTGAGTGTTGGCTGGTGCC
MCAD	F: TTGAGTTCACCGAACAGCAG<br>R: TCCAAGTCCAAGACCTCCAC
ERRα	F: TGCCAATTCAGACTCTGTGC<br>R: CCAGCTTCACCCCATAGAAA
SIRT4	F: GACAAGGTTGATTTTGTGCAC<br>R: TCAAAGGCAGCAACTCTCCAC
TFAM	F: CTTATAGGGCGGAGTGGCAG<br>R: GCTGAACGAGGTCTTTTTGGT
NRF1	F: CACAGAAAAGGTGCTCAAAGGA<br>R: TTTGGGTCACTCCGTGTTCC
CYT.C	F: GAGATGTTCATGCGGCCAG<br>R: ACGTAGTCCTCACCACCAT
COX-IV	F: GAGTGTTGTGAAGAGTGAA<br>R:TCATCTCGGCGAAGCTCTC
CYT-B for mtDNA	F: CCCATCCAACATCTCCGCAT<br>R: GATGAAAAGGCGGTTGAGGC
